# Impact of Crystalline Structural Differences Between α- and β-Chitosan on Their Nanoparticle Formation Via Ionic Gelation and Superoxide Radical Scavenging Activities

**DOI:** 10.3390/polym11122010

**Published:** 2019-12-04

**Authors:** Yattra Jampafuang, Anan Tongta, Yaowapha Waiprib

**Affiliations:** 1 Department of Fishery Products, Faculty of Fisheries, Kasetsart University, Bangkok 10900, Thailand; 2 Division of Biotechnology, School of Bioresources and Technology, King Mongkut′s University of Technology Thonburi, Bangkok 10150, Thailand; 3 Center for Advanced Studies for Agriculture and Food (CASAF), Kasetsart University Institute for Advanced Studies, Kasetsart University, Bangkok 10900, Thailand

**Keywords:** α- and β-chitosan, deacetylation degree, molecular weight, crystalline structure, chitosan nanoparticles, particle size, zeta potential, superoxide radical scavenging activity

## Abstract

α- and β-Chitosan nanoparticles were obtained from shrimp shell and squid pen chitosan with different set of deacetylation degree (%DD) and molecular weight (MW) combinations. After nanoparticle formation via ionic gelation with sodium tripolyphosphate (TPP), the % crystallinity index (%CI) of the α- and β-chitosan nanoparticles were reduced to approximately 33% and 43% of the initial %CI of the corresponding α- and β-chitosan raw samples, respectively. Both forms of chitosan and chitosan nanoparticles scavenged superoxide radicals in a dose-dependent manner. The %CI of α- and β-chitosan and chitosan nanoparticles was significantly negatively correlated with superoxide radical scavenging abilities over the range of concentration (0.5, 1, 2 and 3 mg/mL) studied. High %DD, and low MW β-chitosan exhibited the highest superoxide radical scavenging activity (*p* < 0.05). α- and β-Chitosan nanoparticles prepared from high %DD and low MW chitosan demonstrated the highest abilities to scavenge superoxide radicals at 2.0–3.0 mg/mL (*p* < 0.05), whereas α-chitosan nanoparticles, with the lowest %CI, and smallest particle size (*p* < 0.05), prepared from medium %DD, and medium MW chitosan showed the highest abilities to scavenge superoxide radicals at 0.5–1.0 mg/mL (*p* < 0.05). It could be concluded that α- and β-chitosan nanoparticles had superior superoxide radical scavenging abilities than raw chitosan samples.

## 1. Introduction

Chitosan is a derivative of chitin, which is the second most abundant polymer in Nature after cellulose [1,2,3,4]. Three polymorphic forms (α, β and γ) of chitin have been discovered, which differ in the arrangement of the chains in the crystalline regions; that is, α-chitin has antiparallel chains, while β-chitin has parallel chains for, and γ-chitin is a combination of α- and β-chitin [1,2]. α-Chitosan has been commercially manufactured from shrimp and crab shells and has been widely applied in various fields, whereas β-chitosan has been commonly obtained from squid pens and has a limited number of studies and applications, primarily due to its limited availability [5,6,7,8]. It was reported that the β-chitin crystalline structure was more modified than that of α-chitin after deacetylation, resulting in better properties such as degree of solubility, and swelling capacitity [1,7], which consequently affect their antioxidant activity [7,8]. The antioxidant activities of chitosan have been extensively studied both in vitro and in vivo using different methodologies and are reported to be correlated to its structural characteristics such as degree of deacetylation (%DD), molecular weight (MW), as well as the source of the material [9,10,11]. The physicochemical properties of chitosan end products can be modulated by controlling factors such as chitin source of origin, reaction conditions (concentration, ratios of chitin to alkali, temperature), and extent of the reaction. It is known that hydroxyl, and amino groups in chitosan are key components in eliminating anion radicals such as superoxide and hydroxyl radicals [8,9,10,12]

It has been reported that the amino and hydroxyl groups on chitosan backbone represent target moieties for chemical modifications to improve the aqueous solubility of chitosan since it is soluble only in an aqueous acidic solution, which is a major hurdle for its application [5]. The unique properties of chitosan, such as nontoxicity to humans [13,14,15]; commercial accessibility, biodegradability and biocompatibility [14,15], and high mucoadhesive properties [15,16] make chitosan an excellent choice for nanoparticle assembly. Chitosan-based nanoparticles are commonly obtained by ionic gelation using the multivalent ion tripolyphosphate (TPP) [14,15,16] which possesses a large number of lone-pair electrons and high binding power with materials with empty orbitals [14]. Previous studies have demonstrated the effects of %DD, and MW of chitosan on the characteristics of the resulting nanoparticles [9,17,18,19,20], such as particle size [17,19,20], zeta potential [17,19,20], and crystalline structure [19]. The crystalline structure of chitosan is strongly dependent on its deacetylation process, as well as its chitin polymorphic form [1,21,22,23,24,25]. Ionic cross-linked chitosan nanoparticles are fabricated through electrostatic interactions, in which the amino groups on the backbone interact with polyanionic cross-linking agents offering made-to-order chitosan nanoparticles by modifying the processing parameters, which subsequently influences their functional properties [18]. It is uncertain how differences in polymorphic structure affect the formation of ionic gels [5], which may lead to different antioxidant actions. Previous studies of chitosan polymorphs have revealed differences in crystalline structure between α-, β- and γ-chitosan obtained from various sources [26,27]. The antioxidant properties of chitosan-based nanoparticles have been also widely reviewed [5,28,29]. However, the impact of the crystalline structural differences between α- and β-chitosan on their nanoparticle formation via ionic gelation and their superoxide radical scavenging activities has not yet been compared.

The objective of this study was thus to investigate the effects of crystalline structural differences between α- and β-chitosan on their nanoparticle formation via ionic gelation and their superoxide radical scavenging activities. α-and β-Chitosan nanoparticles were obtained from shrimp shell and squid pen chitosan with different sets of %DD and MW combinations, achieved by controlling the factors such as reaction conditions (concentration, ratios of chitin to alkali, temperature), and extent of the reaction. Physicochemical characteristics of α- and β-chitosan nanoparticles, prepared from corresponding raw α- and β chitosan samples, including particle size, zeta potential, and x-ray diffraction pattern were determined in comparison.

## 2. Materials and Methods

### 2.1. Materials

Dried shrimp shell and squid pen were obtained from a local food processing plant (Samutsakhon, Thailand). *N*-Acetyl-d-glucosamine, sodium tripolyphosphate (TPP), phenazine methosulfate (PMS), β-nicotinamide adenine dinucleotide (NADH), nitroblue tetrazolium, and α-tocopherol were purchased from Sigma Chemical Co. (St. Louis, MO, USA). Acetic acid, hydrochloric acid (HCl) and sodium hydroxide (NaOH) were obtained from VWR (Radnor, PA, USA). Sodium chloride (NaCl), di-potassium hydrogen orthophosphate, and potassium dihydrogen orthophosphate were purchased from Ajax Finechem (North Ryde, NSW, Australia). All the chemicals were of analytical grade.

### 2.2. Preparation of Chitin and Chitosan

α-Chitin and β-chitin were prepared from dried shrimp shell and squid pen, respectively. Dried shrimp shell was demineralized with 1.0 M HCl at a ratio of 1:20 (*w*/*v*) for 2 h at 60 °C and then deproteinized with 2 M NaOH at a ratio of dried shrimp shell to NaOH 1:20 (*w*/*v*) for 2 h at 60 °C, and oven dried at 60 °C. Dried squid pen was deproteinized with 2 M NaOH at a ratio of dried squid pen to NaOH 1:20 (*w*/*v*) for 2 h at 60 °C, and oven dried at 60 °C.

α-Chitosan and β-chitosan were prepared from α-chitin and β-chitin respectively. α-Chitin was deacetylated with 50% NaOH (*w*/*w*) at a range of dried shrimp shell to NaOH ratio 1:30 to 1:20 (*w*/*v*), temperature 120–126 °C and the batch process was repeated 1–4 times, resulting in three α-chitosan products with different %DD and MW combinations, that is, aCS1, aCS2, and aCS3, where: a = α-chitosan, CS = chitosan, 1 = low %DD and high MW, 2 = medium %DD and medium MW, and 3 = high %DD and low MW. β-Chitin was deacetylated with 36–50% NaOH (*w*/*w*) at a range of dried shrimp shell to NaOH ratio 1:30 to 1:20 (*w*/*v*), temperature 120 °C and the batch process was repeated 1–4 times resulting in three β-chitosan products with different %DD and MW combinations, that is, bCS1, bCS2, and bCS3, where: b = β-chitosan, CS = chitosan, 1 = low %DD and high MW, 2 = medium %DD and medium MW, and 3 = high %DD and low MW.

### 2.3. Preparation of Chitosan Nanoparticles

Chitosan nanoparticles were obtained via ionic gelation of chitosan with TPP by a modification of the method of Antoniou et al. [30]. Briefly, α-chitosan and β-chitosan were dissolved in 0.1% (*w*/*v*) acetic acid to obtain 1 mg/mL solution, and the pH was adjusted to 4.8 by 2.0 N NaOH. TPP was dissolved in distilled water to obtain a 1 mg/mL solution. Chitosan nanoparticles were assembled when TPP solution was added dropwise into the chitosan solution at a mass ratio of 5:1 under magnetic stirring (750 rpm) at room temperature. The polymer to crosslinker (TPP) ratio of 5:1 was chosen following preliminary experiment results. Ionic gelation of α-chitosan with TPP resulted in three types of α-nanoparticles, that is, aNP1, aNP2, aNP3, where: a = α-chitosan, NP = nanoparticles, 1 = low %DD and high MW, 2 = medium %DD and medium MW, and 3 = high %DD and low MW. Ionic gelation of β-chitosan with TPP resulting in three types of β-nanoparticles, that is, bNP1, bNP2, bNP3, where: b = β-chitosan, NP = nanoparticles, 1 = low %DD and high MW, 2 = medium %DD and medium MW, and 3 = high %DD and low MW.

### 2.4. Characterisation of Chitosan and Chitosan Nanoparticles

#### 2.4.1. %DD

The %DD of chitosan samples were determined by the first derivative UV spectrophotometry method of Kiang et al. [31]. Firstly, UV visible absorbance spectra of 0.01, 0.02 and 0.03 M acetic acid solutions were obtained from 190–250 nm scanning using a UV-visible spectrophotometer (UV-1700, Shimadzu, Kyoto, Japan). The zero-crossing point (ZCP) was obtained from the intersection of the first derivative absorbance spectra. The standard curve was plotted using 0.005 to 0.035 mg/mL *N*-acetyl-d-glucosamine in 0.01 M acetic acid. The height, *H*, was measured from the ZCP to the first derivative spectra of the standard solutions. The %DD of chitosan was obtained by scanning absorbance spectra of 0.1 mg/mL chitosan in 0.01 M acetic and the concentration of *N*-acetyl-d-glucosamine was determined and the %DD was then calculated.

#### 2.4.2. MW

The MW of chitosan samples were determined according to the method of Jung and Zhao [8]. Chitosan samples were dissolved in 0.1 M acetic acid and 0.2 M NaCl solvent system. An Ubbelohde viscometer (Cannon Instrument Company, State College, PA, USA) was used to measure the efflux time at 25 °C. Intrinsic viscosity (ŋ) was obtained from linear plots of reduced viscosity (ŋ_sp_/C) against concentration (C, g/mL), extrapolating to zero concentration. The MW was assessed using the Mark–Houwink relationship given in Equation (1):

ŋ = K(MW)^a^ (1)

where K = 1.8 × 10^−3^ and a = 0.93.

#### 2.4.3. X-ray Diffraction

X-ray diffraction (XRD) patterns of chitosan and chitosan nanoparticle powder were achieved by X-ray diffractometry (D8 DISCOVER, Bruker, Fällanden, Switzerland) at 40 kV, 40 mA and 2θ with the scan angle from 5° to 60° as described previously [24]. The crystalline index (CI; %) was obtained from the area ratio of the crystal phase to the total phase of crystal and amorphous phase in XRD patterns using Equation (2):

CI_110_ = (*I*_110_ − *I*_am_) × 100/*I*_110_ (2)

where *I*_110_ is the maximum intensity of the lattice diffraction pattern at 20° and *I*_am_, the intensity of amorphous diffraction at 16°.

#### 2.4.4. Particle Size, Polydispersity Index (PDI), and Zeta Potential

The particles size, PDI, and zeta potential of chitosan nanoparticles were done at 25 ± 0.5 °C by dynamic light scattering (DLS) technique using a Zetasizer (Nano-ZS, Malvern Instruments, Malvern, UK).

### 2.5. Superoxide Radical Scavenging Activity

The superoxide scavenging activity of chitosan and chitosan nanoparticles was assessed by the modified method of Xing et al. [32]. The mixture of 0.5–3.0 mg/mL chitosan and chitosan nanoparticles, 30 µM PMS, 338 µM NADH and 72 µM NBT in 0.1 M phosphate buffer (pH 7.4) were incubated at 25 ± 0.5 °C for 5 min and the absorbance was read at 560 nm against a blank. Superoxide radical scavenging activity was calculated by Equation (3):

(3) $$\mathrm{Superoxide}\;\mathrm{radical}\;\mathrm{scavenging}\;\mathrm{activity}\;\left(\%\right)=\left(1-\frac{A_{\mathrm{sample}\;560\;\mathrm{nm}}}{A_{\mathrm{control}\;560\;\mathrm{nm}}}\right)\times 100$$

### 2.6. Statistical Analysis

All data are expressed as means ± standard deviation (SD) from three replicates. One way analysis of variance (ANOVA) were performed, followed by Duncan′s multiple range test for mean comparison. Pearson′s correlation coefficients (r) was used to determine the statistical relationship between two variables.

## 3. Results and Discussion

### 3.1. Characterisation of Chitosan and Chitosan Nanoparticles

#### 3.1.1. %DD and MW of chitosan

**Figure 1:**
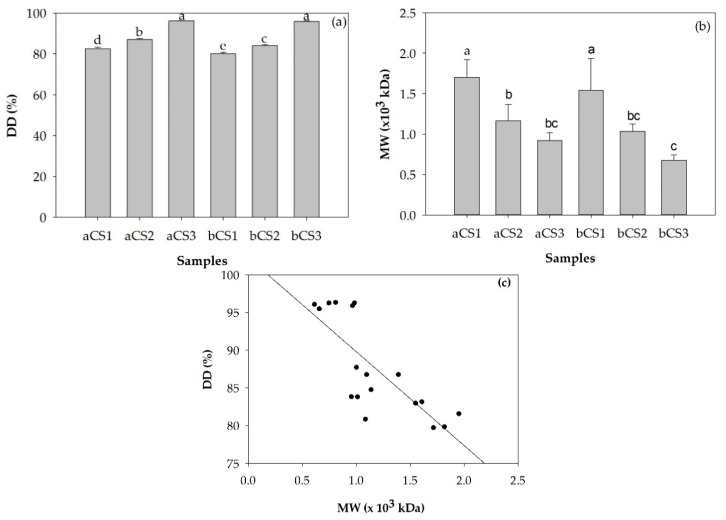
The degree of deacetylation (%DD) (**a**) and molecular weight (MW) (**b**) and Pearson’s correlation coefficient between the %DD and MW (r = −0.779, *p* < 0.01) of α- and β-chitosan samples with different set of %DD and MW combinations (**c**). All the data represent the mean with standard deviation (*n* = 3). Significant differences are indicated by different letters (*p* < 0.05). Where: a = α-chitosan, b = β-chitosan, CS = chitosan, 1 = low %DD and high MW, 2 = medium %DD and medium MW, 3 = high %DD and low MW.

The %DD and MW of α- and β-chitosan samples with different sets of %DD and MW combinations are illustrated in Figure 1a,b, respectively. As shown in Figure 1a, the %DD of either the α- or β-chitosan products were more dependent on the deacetylation process parameters (*p* < 0.05) rather than the chitin crystalline structure. The %DD of α- and β-chitosan were in the range of 82.54–96.16% and 80.12–95.93%, respectively. In general, chitosan product %DD values between 56% and 99%, with an average of 80%, have been reported, dependent on the deacetylation process conditions [25]. The increase in deacetylation process parameters such as concentration, ratios of chitin to alkali, temperature) and extent of the reaction resulted in a higher chitosan end product %DD. Figure 1b illustrates that the MWs of both α- or β-chitosan samples were strongly dependent on deacetylation process parameters (*p* < 0.05) rather than the chitin source of origin. The increase in deacetylation process parameters such as concentration, ratios of chitin to alkali, temperature) and extent of the reaction resulted in lower MW of chitosan end products, accordingly [4]. The MWs of α- and β-chitosan were in the range of 0.9–1.7 (×10^3^) kDa and 0.7–1.5 (×10^3^) kDa, respectively, similar to the reported MW of shrimp shell chitosan of 0.95 to 4.47 (×10^3^) kDa [4]. The Pearson’s correlation result showed that %DD was significantly negative correlated with MW (r = −0.779, *p* < 0.01). It is known that the scission of acetyl moieties occurred at the same time as a reduction of parent chain length during the deacetylation process resulting in a higher %DD of lower MW chitosan products [20].

#### 3.1.2. Crystalline Structure of Chitosan and Chitosan Nanoparticles

**Figure 2:**
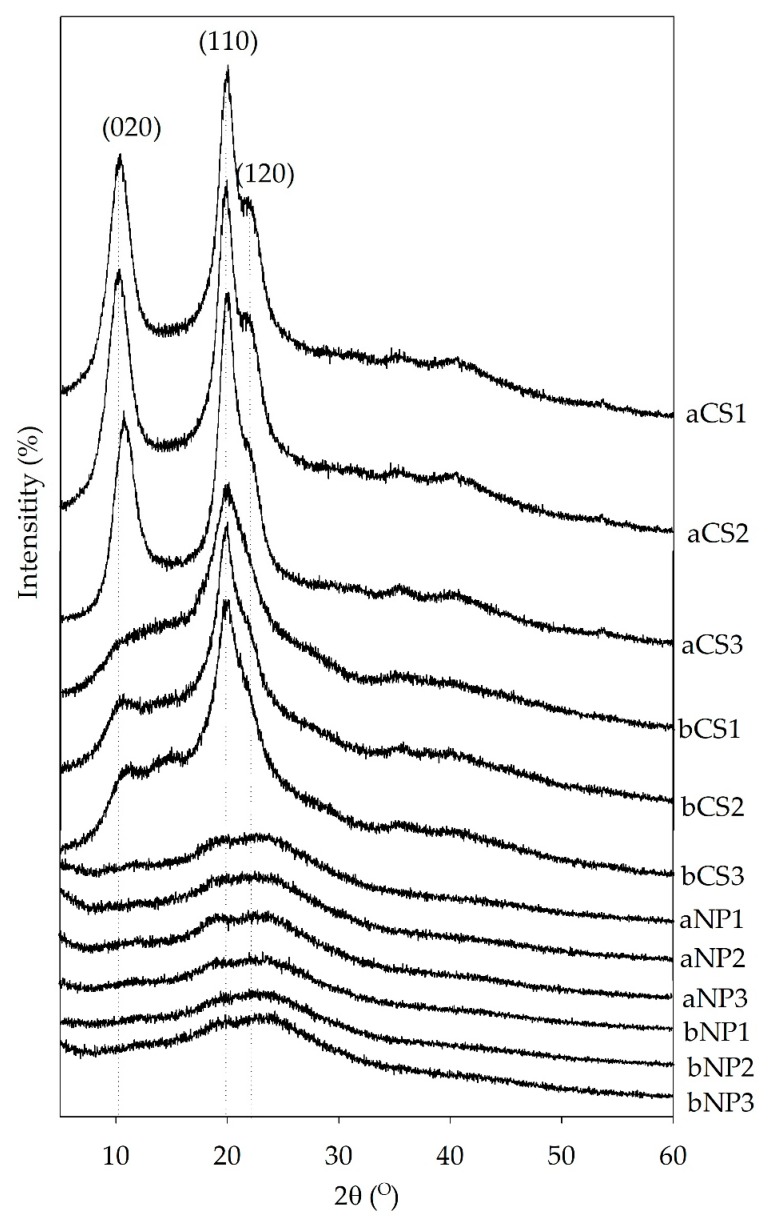
X-ray diffraction patterns of α- and β-chitosan and α- and β-chitosan nanoparticles prepared from chitosan with different set of deacetylation degree (%DD) and molecular weight (MW) combinations, respectively. The analysis was conducted at 40 kV, 40 mA and 2θ with the scan angle from 5° to 60°. Where: a = α-chitosan, b = β-chitosan, CS = chitosan, NP = chitosan nanoparticles, 1 = low %DD and high MW, 2 = medium %DD and medium MW, and 3 = high %DD and low MW. (020), (110) and (120) represent the diffraction peak characteristic at 2θ ≈ 10°, ≈20°, and ≈21°, respectively.

The XRD patterns of chitosan and chitosan nanoparticles are illustrated in Figure 2. The crystalline structure characteristics, in terms of %CI, 2θ, *d*-spacing, and relative intensity (RI, %) of various planes (020, 110 and 120) of the chitosan and chitosan nanoparticles are summarized in Table 1. Figure 2 and Table 1 show the presence of two strong characteristic peaks at 2θ around 10°–11° (amine I “–N–CO–CH_3_” of chitosan), 19°–20° (amine II “–NH_2_” of chitosan), and a shoulder in 21°, in agreement with previous reports, which correspond to the (110), (020), and (120) planes, respectively [7,24,29,33]. The XRD patterns suggested an α- and β-chitosan crystalline structure as reported previously [25,34]. Similar to α-chitosan XRD patterns, the β-chitosan XRD pattern exhibited characteristic crystalline peaks at 2θ ≈ 20°. However, compared to α-chitosan XRD patterns, the crystalline peaks at 2θ ≈ 10° of β-chitosan disappeared due to the weak intermolecular force [35], due to the absence of hydrogen bonds between two inter-sheets of its parallel arrangement [8]. It was obvious that a decrease in %relative intensity of the (110) and (020) planes of chitosan nanoparticles and shift into the (120) plane occurred, reflecting that native α- and β-chitosan were successfully transformed into nanoparticles. The peak near 10° suddenly disappeared, while the peak near 20° became broad and weaker, indicating an amorphous state of their crystalline structures and a substantial reduction of intramolecular hydrogen bonds [22,23,33].

It is known that the crystalline structure of chitosan is destroyed by ionic cross-linking interactions between the amino groups on chitosan and TPP [14,30]. Depolymerization reactions could have occurred favorably through the crystalline domains of the biopolymer, resulting in a decrease in the intensity of the characteristic peaks of chitosan [36]. Moreover, the *d*-spacing values of the *N*-acetyl-d-glucosamine portion and *N*-glucosamine portion were in agreement with previous reports [26,36].

As shown in Table 1, the %CI values of α-chitosan products were significantly higher than thise of β-chitosan products (*p* < 0.05), as it is known that %CI of α-chitin is higher than β-chitin [1,8]. The %CI of α- and β-chitosan nanoparticles were significantly lower than those of corresponding chitosan raw materials (*p* < 0.05), which is in agreement with previous findings [5]. The average %CI values were 67.26%, and 52.12% for the α- and β-chitosan samples, respectively, whereas the average %CI values were 22.29% and 22.60% for the α- and β-chitosan nanoparticles, respectively. %CI values ~30–80% and ~10–30% were reported for native chitosan and chitosan nanoparticles [5,33]. It is obvious that %CI of chitosan nanoparticles prepared from either α-, and β-chitosan were not significantly different (*p* ≥ 0.05), except for the aNP2 sample which showed little variation. To some extent, the %CI of α- and β-chitosan was positively correlated with the %DD of chitosan, while it was negatively correlated with MW, similar to a previous report [7]. The reduction in %CI of chitosan nanoparticles was a result of a decrease in the intensity of chitosan characteristic peaks at 2θ ~ 20° as described above [33].

#### 3.1.3. Particle Size, Polydispersity Index (PDI) and Zeta Potential of Chitosan Nanoparticles

**Figure 3:**
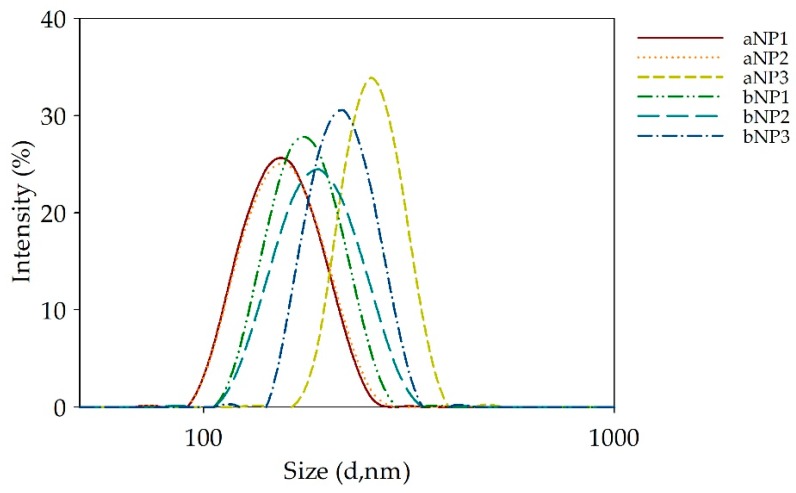
Particles size distribution by intensity of α- or β-chitosan nanoparticles prepared from chitosan with different set of deacetylation degree (%DD) and molecular weight (MW) combinations respectively. Where: a = α-chitosan, b = β-chitosan, NP = chitosan nanoparticles, 1 = low %DD and high MW, 2 = medium %DD and medium MW, 3 = high %DD and low MW.

**Figure 4:**
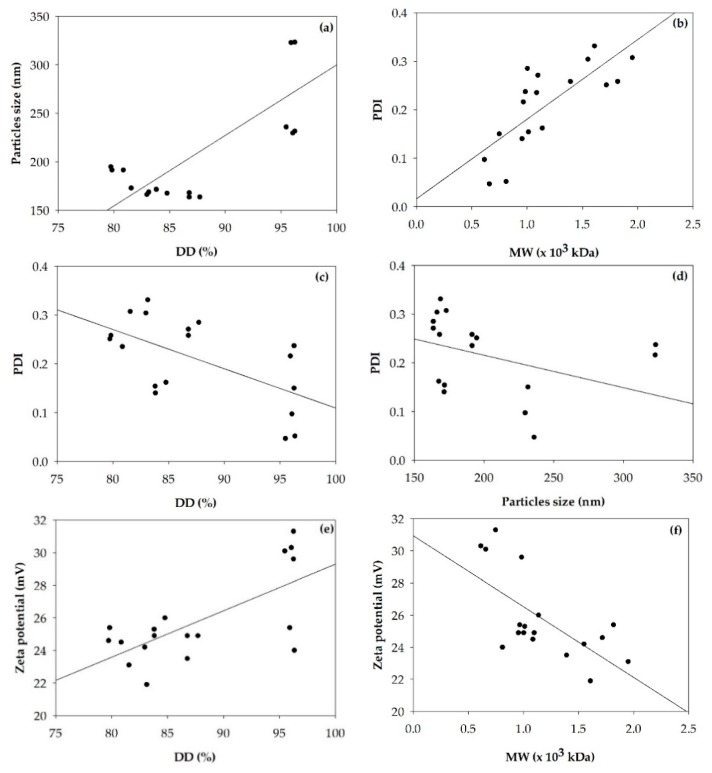
Pearson’s correlation coefficient between particle size of chitosan nanoparticles and deacetylation degree (%DD) of chitosan (r = 0.770, *p* < 0.01) (**a**), PDI of chitosan nanoparticles and molecular weight (MW) of chitosan (r = 0.766, *p* < 0.01) (**b**), PDI and %DD (r = −0.602, *p* < 0.01) (**c**), PDI and particle size (r = −0.469, *p* < 0.05) (**d**), zeta potential of chitosan nanoparticles and %DD (r = 0.686, *p* < 0.01) (**e**), zeta potential and MW (r = −0.662, *p* < 0.01) (**f**).

The particle sizes of chitosan nanoparticles formed via ionic gelation are demonstrated in Figure 3, and summarized in Table 2, where the PDI, and zeta potential of chitosan nanoparticles formed via ionic gelation are also listed. Pearson′s correlations between particle size, PDI, zeta potential of chitosan nanoparticles and %DD and MW of chitosan are presented in Figure 4.

The sizes of α- and β-chitosan nanoparticles were in the range of 165 to 332 nm and 170 to 232 nm, respectively. Figure 4a demonstrates that the particle sizes of α-and β-chitosan nanoparticles were significantly and positively correlated with the %DD of chitosan (r = 0.770, *p* < 0.01). The higher %DD of chitosan resulted in the bigger particle size of either α- or β-chitosan nanoparticles. This suggested that the particle size could be related to the −NH_2_ groups available in the chitosan polymer chains. Higher %DD chitosan may indicate a stronger protonation of the –NH_2_ moiety leading to stronger molecular repulsion, which made the chitosan polymer chain stretch larger and resulted in a larger particle size [17], while a lower MW might not translate into smaller nanoparticles due to a reduced chain entanglement tendency [17]. Higher MW chitosan chains may be able to entangle with each other into more compact particles than those of lower MW chitosan [19].

The PDI values of α- and β-chitosan nanoparticles were in the range of 0.168–0.314 and 0.098–0.248, respectively. In case of nanoparticles, a PDI below 0.3 is desired, since values higher than 0.3 indicate low uniformity, being an indication of aggregation [37]. Figure 4b demonstrates that the PDIs of α-and β-chitosan nanoparticles were significantly and positively correlated with the MW of the chitosan (r = 0.766, *p* < 0.01), but negatively correlated with %DD (r = −0.602, *p* < 0.01) as seen in Figure 4c, and particle size (r = −0.469, *p* < 0.05) as seen in Figure 4d. The higher MW of chitosan resulted in the higher PDI of either the α- or β-chitosan nanoparticle size distribution. From Table 2, only the aNP1 sample showed a PDI greater than 0.3 which could have resulted from using high MW chitosan, which may be able to entangle into a higher polydispersity sample with multiple particle size populations [37].

The zeta potentials of α- and β-chitosan nanoparticles were in the range of 23.07–26.33 and 24.83–30.57 mV, respectively. Figure 4e shows that the zeta potentials of the α- and β-chitosan nanoparticles were significantly and positively correlated with %DD of chitosan (r = 0.686, *p* < 0.01, Figure 4e), but negatively correlated with the chitosan MW (r = −0.662, *p* < 0.01), as seen in Figure 4f. Nanoparticle dispersion zeta potential values of ±20–30 mV and ±30 mV are considered stable and very stable, respectively [38]. From Table 2, the bNP3 sample showed the highest zeta potential value, with a value greater than 30 mV, indicating it was the most stable sample among all samples studied. The reduction in zeta potential of chitosan nanoparticles prepared from higher MW chitosan might be a reason for the lower %DD when compared to that of lower MW chitosan with higher %DD [19].

The particle sizes of aNP2 and bNP2 were the smallest, with PDIs lower than 0.3 indicating that there might be an optimal %DD and MW for nanoparticle formation. It is clear that the %DD, and MW of chitosan exerted combined effects on the chitosan nanoparticle characteristics [18].

### 3.2. Superoxide Radical Scavenging Activities of Chitosan and Chitosan Nanoparticles

**Figure 5:**
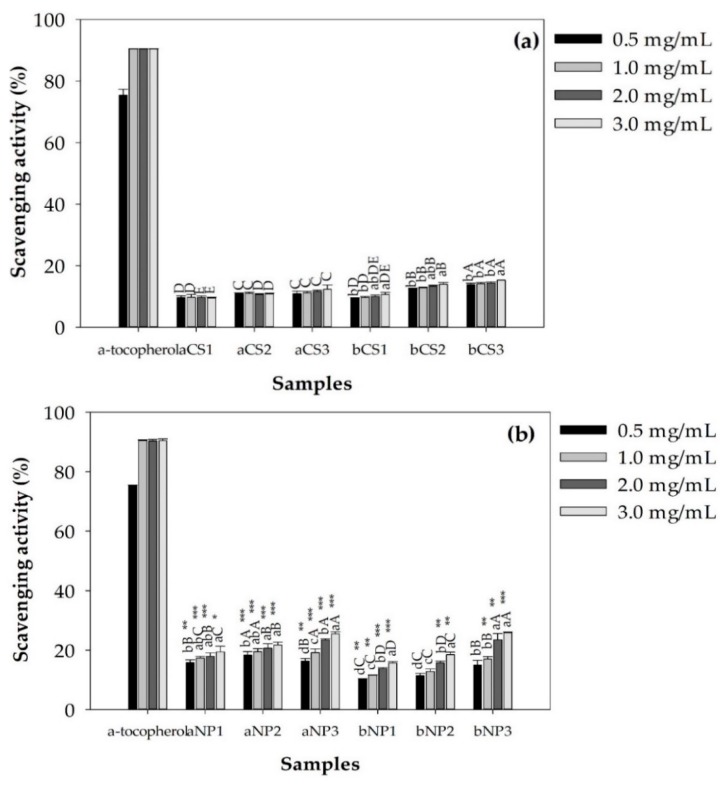
Superoxide radical scavenging activities of α- and β-chitosan (**a**) and α- and β-chitosan nanoparticles (**b**). Values were obtained from three independent experiments. Different lowercase letters (a,b,c…) on the graphics at the same sample indicate significant difference (*p* < 0.05) between the results. Different capital letter (A,B,C…) on the graphics at the same concentration indicate significant difference (*p* < 0.05) between the results. (*) refers to statistically significant difference (*p* < 0.05), (**) refers to statistically significant difference (*p* < 0.01), and (***) refers to a statistically significant difference (*p* < 0.001) when compared to chitosan in (a). α-Tocopherol was used as a standard and was not compared with all samples studied. Where: a = α-chitosan, b = β-chitosan, CS = chitosan, NP = chitosan nanoparticles, 1 = low %DD and high MW, 2 = medium %DD and medium MW, 3 = high %DD and low MW.

**Figure 6:**
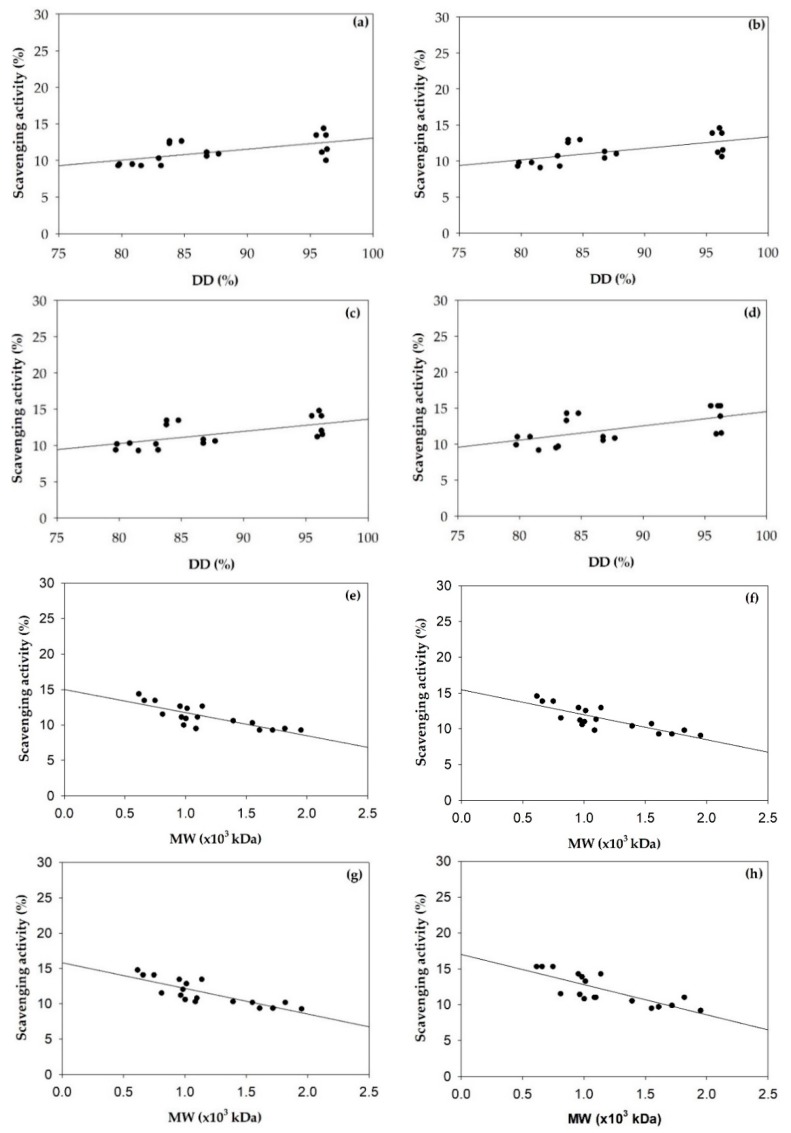
Pearson’s correlation coefficient between the deacetylation degree (%DD) of chitosan and superoxide radical scavenging activities of α- and β-chitosan at concentration of 0.5 mg/mL (r = 0.594, *p* < 0.01) (**a**), 1 mg/mL (r = 0.599, *p* < 0.01) (**b**), 2 mg/mL (r = 0.604, *p* < 0.01) (**c**) and 3 mg/mL (r = 0.596, *p* < 0.01) (**d**) and between the molecular weight (MW) of chitosan and superoxide radical scavenging activities of chitosan at concentration of 0.5 mg/mL (r = −0.805, *p* < 0.01) (**e**), 1 mg/mL (r = −0.822, *p* < 0.01) (**f**), 2 mg/mL (r = −0.815, *p* < 0.01) (**g**) and 3 mg/mL (r = −0.787, *p* < 0.01) (**h**).

The superoxide radical scavenging activities of α- and β-chitosan and chitosan nanoparticles at concentrations of 0.5, 1, 2 and 3 mg/mL are given in Figure 5, indicating that α- and β-chitosan and α- and β-chitosan nanoparticles scavenged superoxide radicals in a dose-dependent manner. Figure 5a demonstrates that the superoxide radical scavenging activities of high %DD and low MW chitosan was more pronounced than that of low %DD and high MW chitosan. The effects of %DD of α- and β-chitosan on the superoxide radical scavenging activities of chitosan were estimated by Pearson’s correlation coefficients presented in Figure 6a–d, as well as the effects of MW of α- and β-chitosan on superoxide radical scavenging activities of chitosan were estimated by Pearson’s correlation coefficients and are presented in Figure 6e–h. It was obvious that %DD of α- and β-chitosan were significantly positively correlated with the superoxide radical scavenging activities of chitosan, while the MWs of α- and β-chitosan were significantly negatively correlated with the superoxide radical scavenging activities of chitosan. Previous reports showed that enzyme partially degraded products of chitosan with low MW had higher antioxidant activity than chitosan with high MW [11]. High MW chitosan has compact structure due to the stronger hydrogen bonds, which weakens the activity of the hydroxyl and amino groups. However, low MW has a less compact structure, so the effect of the intramolecular hydrogen bonds is weak and more free hydroxyl and amino groups [9]. It is apparent that β-chitosan had the abilities to eliminate superoxide radicals higher than α-chitosan (*p* < 0.05) and β-chitosan at the highest %DD, that is, bCS3 sample, showed the highest ability to eliminate superoxide radicals. (*p* < 0.05). Superoxide radical is a zwitterionic radical, which could react with free hydroxyl and amino groups in chitosan, then superoxide radical was scavenged by this reaction [32]. It could be extrapolated to comment that is bCS3 sample with the highest %DD, resulting in higher number of active hydrogen which could be a positive factor that affects the scavenging activity against superoxide radical as reported previously [39,40]. Higher degree of solubility with less crystallinity was reported for β-chitosan, resulting in better functional properties than α- chitosan with similar MW and %DD [8].

**Figure 7:**
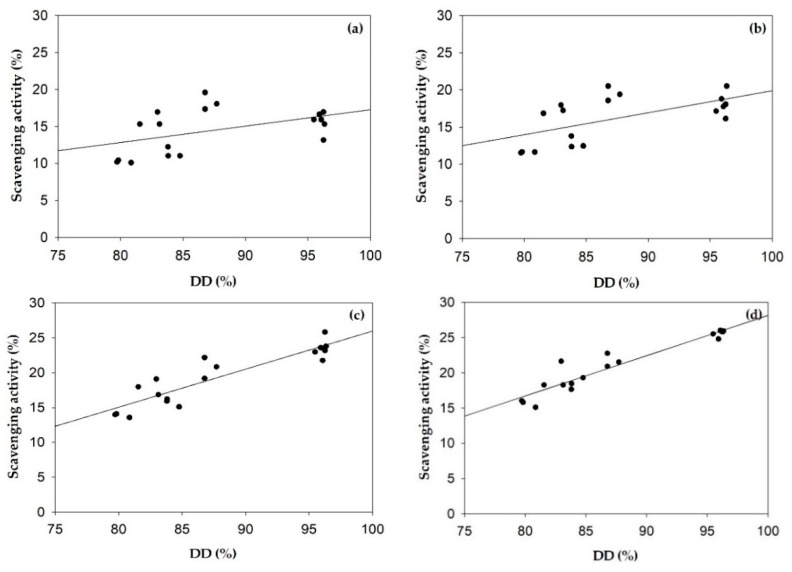
Pearson’s correlation coefficient between the deacetylation degree (%DD) of chitosan and superoxide radical scavenging activities of α- and β-chitosan nanoparticles at concentration of 0.5 mg/mL (r = 0.477, *p* < 0.05) (**a**), 1 mg/mL (r = 0.607, *p* < 0.01) (**b**), 2 mg/mL (r = 0.905, *p* < 0.01) (**c**) and 3 mg/mL (r = 0.952, *p* < 0.01) (**d**).

The superoxide radical scavenging activities of α- and β-chitosan nanoparticles are illustrated in Figure 5b. The effects of %DD of α- and β-chitosan on superoxide radical scavenging activities of α- and β-chitosan nanoparticles were estimated by Pearson’s correlation coefficients presented in Figure 7. It was apparent that %DD of α- and β-chitosan were significantly positive correlated with superoxide radical scavenging activities of α- and β-chitosan nanoparticles. At 2.0–3.0 mg/mL α- and β-chitosan nanoparticles prepared from the highest % DD and lowest MW; that is, aNP3, and bNP3 samples showed the highest abilities to eliminate superoxide radicals (*p* < 0.05). This may result from the higher zeta potential (*p* < 0.05) as seen in Table 2, and Figure 4e, suggesting that higher %DD chitosan produced higher zeta potential chitosan nanoparticles, resulting in higher magnitude of the electrostatic or charge repulsion/attraction between particles from amino groups at the C2 positions similar to previous findings [9,17]. In particular, the efficient antioxidant activity may be due to the high ratio of electrons remaining at the surface, and thus available to scavenge free radicals [9,41]. However, at the lower range of concentrations used, that is, 0.5–1.0 mg/mL, α-chitosan nanoparticles prepared from medium %DD, and medium MW, that is, the aNP2 sample, showed the highest ability to eliminate superoxide radicals (*p* < 0.05). This may result from the lowest% CI, and smallest particle size (*p* < 0.05) of the aNP2 sample shown in Table 1 and Table 2, respectively, indicating loosely bonding bonds within the molecules with greater surface area allowing more ability to scavenge radicals [9].

**Figure 8:**
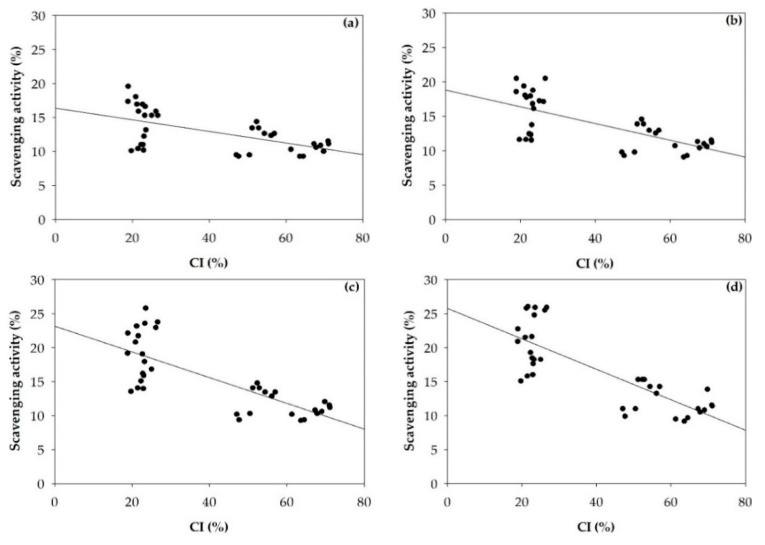
Pearson’s correlation coefficient between the crystallinity index (%CI) and superoxide radical scavenging activities of α- and β-chitosan and α- and β-chitosan nanoparticles at concentration of 0.5 mg/mL (r = −0.580, *p* < 0.01) (**a**), 1 mg/mL (r = −0.688, *p* < 0.01) (**b**), 2 mg/mL (r = −0.765, *p* < 0.01) (**c**) and 3 mg/mL (r = −0.805, *p* < 0.01) (**d**).

In addition, the superoxide scavenging activities of α- and β-chitosan (Figure 5a) were significantly lower compared with their nanoparticles (Figure 5b) except for bNP2 at 0.5–1 mg/mL. Previous report stated that the antioxidant activity of chitosan increased in its nano form [28]. This may result from the less compact structure of chitosan nanoparticles than in the chitosan raw material, as indicated by %CI (Figure 8). Low %CI indicates loosely bonding bonds within the molecules with greater surface area allowing more ability to scavenge radicals [9] as well as the hydrogen bonds in α- and β-CS chain were destroyed to supply more free hydroxyl and amino groups [5].

The effects of crystalline structure in term of %CI on superoxide radical scavenging activities of α- and β-chitosan and α- and β-chitosan nanoparticles were estimated by the Pearson′s correlation coefficients presented in Figure 8. It is obvious that %CI of α- and β-chitosan and chitosan nanoparticles were significantly negatively correlated with superoxide radical scavenging abilities at the range of concentrations studied, showing similar patterns.

## 4. Conclusions

The highlight of this work was the preparation of α- and β-chitosan nanoparticles from shrimp shell and squid pen chitosan products with different sets of molecular weight (MW) and deacetylation degree (%DD) combinations in order to relate their different crystalline structures to their antioxidant properties on superoxide radicals. The effects of crystalline structural differences between α- and β-chitosan on their nanoparticle formation via ionic gelation were obvious, in terms of %CI, that is, a decrease in %relative intensity of the (110) and (020) planes of chitosan nanoparticles and a shift to the (120) plane reflected that native α- and β-chitosan successfully formed α- and β-chitosan nanoparticles. The characteristics of these chitosan nanoparticles, such as particle size, PDI, zeta potential, and %CI were strongly affected by not only the chitin sources of origin, but also the chitosan raw sample characteristics, such as %DD, and MW. α- and β-Chitosan samples and α- and β-chitosan nanoparticles scavenged superoxide radicals in a concentration-dependent manner. β-Chitosan with higher %DD, and lower MW showed higher superoxide radical scavenging ability. This study suggests that chitosan nanoparticles, either in the α- or β-form, with small particle size, and high zeta potential could be preferentially obtained from chitosan with defined %DD, and MW, in order to achieve high superoxide radical scavenging ability. This study also suggested that more attention should be paid on %DD and MW and chitin source of raw chitosan samples in order to achieve rationally designed nanoparticles.

## Figures and Tables

**Table 1:** Crystallinity index (CI, %), 2θ(°), *d*-spacing (*d*, Å), and relative intensity (RI, %) of various planes (020, 110 and 120) * appeared in X-ray diffraction patterns of α- and β-chitosan and α- and β-chitosan nanoparticles prepared from chitosan with different set of deacetylation degree (%DD) and molecular weight (MW) combinations, respectively.

Samples	CI		(020)			(110)			(120)	
	(%)	2θ(°)	*d* (Å)	RI (%)	2θ(°)	*d* (Å)	RI (%)	2θ(°)	*d* (Å)	RI (%)
aCS1	63.12 ± 1.67^c^	10.2	8.63	80	20.1	4.42	100	21.9	4.05	68
aCS2	68.04 ± 0.89^b^	10.3	8.53	77	20.0	4.43	100	21.8	4.07	65
aCS3	70.63 ± 0.71^a^	10.6	8.28	68	19.9	4.46	100	21.8	4.08	61
bCS1	48.42 ± 1.82^f^	10.2	8.64	44	19.9	4.46	100	-	-	-
bCS2	55.81 ± 1.32^d^	10.6	8.33	44	19.9	4.46	100	21.8	4.08	70
bCS3	52.13 ± 0.86^e^	11.3	7.86	46	20.0	4.44	100	21.1	4.20	80
aNP1	23.63 ± 1.23^g^	11.7	7.55	73	19.4	4.58	95	22.6	3.94	100
aNP2	19.55 ± 1.15^h^	12.0	7.38	75	19.84	4.47	99	22.4	3.96	100
aNP3	23.70 ± 2.73^g^	11.8	7.49	76	19.1	4.65	98	22.6	3.93	100
bNP1	21.36 ± 1.59^gh^	9.2	9.55	69	19.0	4.67	97	22.0	4.03	100
bNP2	22.70 ± 0.37^g^	12.2	7.26	74	19.8	4.49	97	22.6	3.93	100
bNP3	23.74 ± 2.29^g^	12.7	6.97	74	19.8	4.48	99	22.6	3.93	100

%CI was calculated by equation: (*I*_110_ − *I*_am_)/*I*_110_ × 100, where I_110_ was the maximum intensity of the reflection (110) at 2θ = 20° and *I*_am_ was the intensity of the amorphous diffraction in the same unit at 2θ = 16°. The data of %CI represent the mean with standard deviation. Significant differences are indicated by different letters in the same column (*p* < 0.05). Where: a = α-chitosan, b = β-chitosan, NP = chitosan nanoparticles, 1 = low %DD and high MW, 2 = medium %DD and medium MW, 3 = high %DD and low MW. * (020), (110) and (120) represent the diffraction peak characteristic at 2θ ≈ 10°, ≈20°, and ≈21° respectively.

**Table 2:** Particles size, polydispersity index (PDI) and zeta potential of α- or β-chitosan nanoparticles prepared from chitosan with different set of deacetylation degree (%DD) and molecular weight (MW) combinations respectively.

Samples	Particles Size (nm)	PDI *	Zeta Potential (mV)
aNP1	169.23 ± 3.33^d^	0.314 ± 0.015^a^	23.07 ± 1.15^c^
aNP2	165.03 ± 2.57^d^	0.271 ± 0.014^a^	24.43 ± 0.81^bc^
aNP3	332.20 ± 15.85^a^	0.168 ± 0.101^bc^	26.33 ± 2.91^b^
bNP1	192.50 ± 1.91^c^	0.248 ± 0.012^ab^	24.83 ± 0.49^bc^
bNP2	170.13 ± 2.29^d^	0.152 ± 0.011^c^	25.40 ± 0.56^bc^
bNP3	232.30 ± 3.27^b^	0.098 ± 0.052^c^	30.57 ± 0.64^a^

* PDI = polydispersity index. All the data represent the mean with standard deviation (n = 3). ^a,b,c,…^ The different letters present statistical difference (*p* < 0.05). Where: a = α-chitosan, b = β-chitosan, NP = chitosan nanoparticles, 1 = low %DD and high MW, 2 = medium %DD and medium MW, and 3 = high %DD and low MW.
